# How effective are three methods of teaching oral hygiene for adolescents undergoing orthodontic treatment? The MAHO protocol: an RCT comparing visual, auditory and kinesthetic methods

**DOI:** 10.1186/s13063-021-05093-z

**Published:** 2021-02-15

**Authors:** Alisée Le Fouler, Sylvie Jeanne, Olivier Sorel, Damien Brézulier

**Affiliations:** 1 Univ Rennes, CHU Rennes, Pôle Odontologie, 2 av. du Professeur Léon Bernard, Bât. 15, 35043 Rennes Cedex, France; 2 Univ Rennes, ISCR, CNRS—UMR 6226, CHU Rennes, Pôle Odontologie, 2 av. du Professeur Léon Bernard, Bât. 15, 35043 Rennes Cedex, France

**Keywords:** Orthodontic treatment, Oral health, Education, Auditory learning, Visual learning, Kinesthetic learning, Study protocol, Guideline, Prevention

## Abstract

**Background:**

Fixed orthodontic appliances hamper oral hygiene procedures. The consequences are gingivitis and white spot lesions. Fifty to 70% of patients treated with braces encounter these problems. Their care in the USA represents an annual cost of five hundred million dollars. Initial education and motivation for oral hygiene depend on two categories of factors: firstly, practical prophylactic measures (instruments and medication, professional care) and secondly, the educational component: choice of communication technique, frequency, and nature of hygiene instructions. This trial aims to study this last component. Its main objective is to compare three methods’ effectiveness of oral hygiene education in adolescent patients treated with braces in terms of biofilm (plaque) control. The secondary objectives are the evaluation of these methods’ effectiveness regarding gingival inflammation and the maintenance of hygiene during the first 6 months of treatment.

**Methods:**

This study is a prospective randomized controlled trial of superiority. It evaluates the effectiveness of three hygiene education techniques. A total of 90 patients from the University Hospital Center of Rennes will be randomized into 3 parallel groups with a 1:1:1 ratio. Each will benefit from a different educational method: oral and/or practical. The main outcome will be the average plaque index for each group after 6 months of treatment. Additional outcomes will be the average gingival index for each group and the plaque and gingival indices over 6 months.

**Discussion:**

The effectiveness of preventive procedures for optimizing oral hygiene during orthodontics is based on ambiguous literature. As a result, it is difficult to draw conclusions and to translate them into everyday practice. Sixty-eight percent of the orthodontists support the development of guidelines for education. The aim of this study is to standardize methods of oral hygiene education during orthodontic fixed treatment. The purpose of this study would be to provide practitioners with a concrete education program through guidelines dedicated to the method having the best results.

**Trial registration:**

ClinicalTrials.gov NCT04444154. Registered on 22 June 2020. SI CNRIPH ID 8011N° 20.04.27.58337. Registered on 29 July 2020

## Administrative information

**Table:** 

Title {1}	Auditory versus visual vs kinesthetic learning hygiene education in adolescents with orthodontic braces: a randomized controlled trial. Trial acronyme: MAHO
Trial registration {2a and 2b}.	This trial is registered with the ClinicalTrial.gov under number NCT04444154, registration date 06/22/2020. Réf. CHU de Rennes: 35RC20_8945_MAHO N° ID-RCB: 2020-A00967-32 SI CNRIPH ID 8011N° 20.04.27.58337
Protocol version {3}	Version 1.1, 06/05/2020
Funding {4}	This study is funded by the dedicated research fund of the research and innovation department of the Rennes hospital. The hygiene kits are offered by the company Oral B.
Author details {5a}	1. Alisée Le Fouler: Univ Rennes, CHU Rennes, Pôle Odontologie, 2 Rue Henri Le Guillou, 35033 Rennes Cedex, France 2. Sylvie Jeanne: Univ Rennes, ISCR, CNRS—UMR 6226, CHU Rennes, Pôle Odontologie, 2 av. du Professeur Léon Bernard, Bât. 15, 35043 Rennes Cedex 3. Olivier Sorel: Univ Rennes, CHU Rennes, Pôle Odontologie, 2 av. du Professeur Léon Bernard, Bât. 15, 35043 Rennes Cedex 4. Damien Brézulier*: Univ Rennes, ISCR, CNRS—UMR 6226, CHU Rennes, Pôle Odontologie, 2 av. du Professeur Léon Bernard, Bât. 15, 35043 Rennes Cedex
Name and contact information for the trial sponsor {5b}	Dr METTEN Marie-Astrid, Service d’épidémiologie et de santé publique, CHU de Rennes – Hôpital de Pontchaillou, 2 rue Henri Le Guilloux, 35 033 Rennes Cedex 9. Tel: 02.99.28.93.32 – Email: Marie-astrid. METTEN@chu-rennes.fr
Role of sponsor {5c}	This study is sponsored by the firm Oral B (Proster & Gamble) which offers all participants a dental hygiene kit. This kit consists of an electric toothbrush and tubes of toothpaste. This commercial firm does not intervene in any case on the inclusion of patients or the processing of data.

## Introduction

### Background and rationale {6a}

The medical relevance of orthodontic treatment no longer needs to be demonstrated today. The aim of orthodontic treatment is to restore oro-facial health, i.e., a “state of physical, mental and social well-being” as defined by the WHO [1]. The Oral Health-related Quality of Life (OHrQoL) index is a multidimensional concept that includes a subjective assessment of oral health, functional well-being, emotional well-being, expectations and satisfaction with care, and sense of self [2]. Several scores are used to establish the OHrQoL index. The lower is the score, the better is the quality of life. This index is negatively impacted by malocclusions in both adolescents and adults, making sense to begin orthodontic treatment [3].

As with any medical treatment, the balance between the expected benefits and the involved risks must remain positive. With bonded braces, major iatrogenic effects are gingivitis and enamel demineralization [4, 5]. These adverse consequences affect 50 to 70% of patients treated with braces [6]. Gingivitis, a reversible inflammatory disease of the superficial periodontium (gums), and enamel demineralization are both linked to the accumulation of bacterial biofilm induced by orthodontic braces [7, 8]. Indeed, these last lead to a change in the quantity and quality of the bacterial flora [9, 10]. From a quantitative point of view, the colonization of hard surfaces, such as braces, proves to be faster [11]. In addition, orthodontic appliances create a highly retentive surface. From a qualitative point of view, the appliance bonding results in an increase in Gram + and Gram − aggressive bacteria such as *Streptococcus mutans*, *Lactobacillus* spp., *Porphyromonas gingivalis*, *Tannerella forsythia*, and *Treponema denticola* [12]. These are closely associated with the development of carious lesions and periodontal disease [13].

Regardless of gender and age, a significant relation has already been demonstrated between bad oral hygiene measures and carious lesion formation [14]. Moreover, without early treatment, 15% of patients with a dental appliance will suffer of irreversible lesions of both iatrogenic effects. In the USA, these treatments cost the health system five hundred million dollars a year [15]. In this macro-economic context, a real benefit should be expected from multi-professional consultation. Regarding the literature, many preventive strategies have been developed to optimize oral hygiene during treatment. Unfortunately, study protocols are at high risk of bias: problems with randomization, maintenance of blindness, and short-term follow-up [16, 17]. It is therefore difficult to draw evidence-based conclusions and to translate them into everyday practice. Even today, no guideline is available for the orthodontists to standardize oral hygiene education during brace treatment. This problem appears to be international. A recent survey reports that 68% of the Dutch orthodontists are in favor of developing such recommendations [18].

Initial hygiene education and motivation as well as the maintenance of a minimal plaque index throughout orthodontic treatment depend on two factors: first, practical prophylactic measures: instruments and medication and professional care, and secondly, the educational component: choice of communication technique, frequency, and nature of hygiene instructions.

The main objective of this research is to determine the most effective method of teaching hygiene to limit the adverse effects caused by orthodontic braces in adolescents.

### Objectives {7}

This trial aims to study the educational component. The main objective is to compare auditory vs visual vs kinesthetic methods’ effectiveness of oral hygiene education in adolescent patients treated with braces in terms of biofilm control. The secondary objectives are the evaluation of the effectiveness of these methods regarding gingival inflammation and the maintenance of hygiene during the first 6 months of treatment. The trial will evaluate the hypothesis that the plaque index after 6 months of treatment will be the lowest in the visual + kinesthetic learning method group. The goal of this study would be to provide practitioners with guidelines dedicated to the method having obtained the best results.

### Trial design {8}

**Fig. 1:**
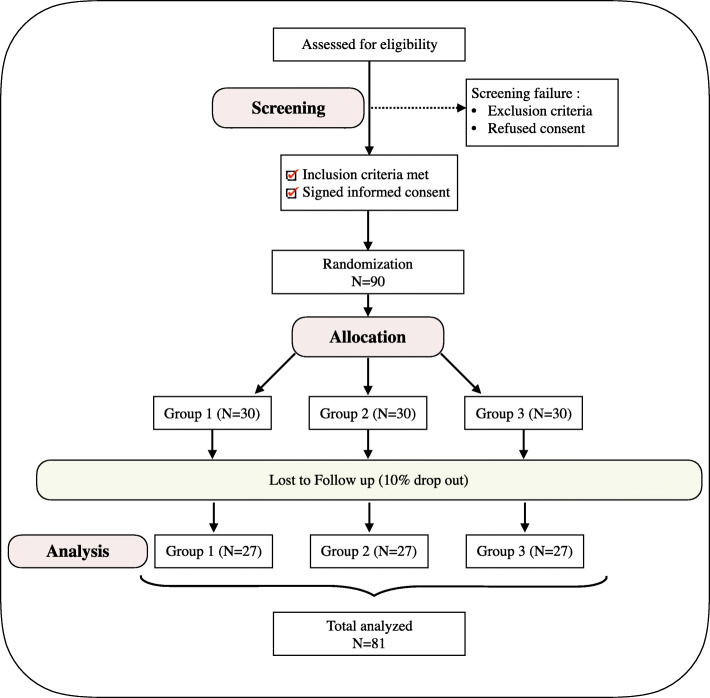
Trial protocol flow chart

This study is a 6-month, monocentric, randomized controlled trial (Fig. 1). Patients from the University Hospital Center of Rennes will be randomized into three groups running in parallel and an allocation ratio of 1:1:1. Each will benefit from a different educational method: oral and/or practical. This study adheres to the Consolidated Standards of Reporting Trials (CONSORT) 2010 Statement.

## Methods: participants, interventions, and outcomes

### Study setting {9}

As it is a monocentric trial, all patients will come from the University Hospital Center of Rennes. The orthodontic department of this Hospital Center is composed of two full-time hospital practitioners and 10 residents in orthodontics. Consultations run from Monday morning to Friday evening. More than 1000 patients are currently undergoing orthodontic treatment in this department. The patients will be treated by all ten residents under the supervision of the professors or by one of these professors. All residents will participate in the study, and each of them will receive a workbook describing the study protocol from the inclusion phase to the exit of the study for each patient. Also, to acquire the follow-up routine, role-play sessions will be done with the residents.

### Eligibility criteria {10}

To be included in the study, patients must meet the following criteria:

- Aged from 11 to 17
- Stable adolescent or young adult or adult dentition
- Requiring fixed orthodontic treatment without extraction (except wisdom teeth) at least at the maxillary arch
- Affiliated himself or through his parents with a social security scheme

Exclusion criteria are as follows:

- Periodontal disease such as increased periodontal clinical attachment loss
- Increased depth of the carious lesion
- Smokers
- Prosthetic crown or restoration on a maxillary incisor
- Systemic disease, syndrome or cleft palate
- Dental structural abnormality (e.g., fluorosis, MIH, amelogenesis imperfecta...)
- Long-term medications influencing periodontal health (corticosteroids, anti-epileptics...)
- Physical or mental disabilities avoiding autonomous tooth brushing
- Refusal to use products and instruments prescribed as part of the study
- Dental agenesis
- Low skills on mastering the French language

### Who will take informed consent? {26a}

Patients and parents will receive an information letter describing the objectives, the process of the study, and the benefits and risks involved. They will be required to sign an informed consent prior to beginning the trial. This consent will be collected by the principal investigator (DB).

### Additional consent provisions for collection and use of participant data and biological specimens {26b}

Not applicable.

## Interventions

### Explanation for the choice of comparators {6b}

In this study, several learning styles will be compared. The methods are those described by Barbe. These are commonly used in pedagogy and teaching and identified by the acronym VAK: visual, auditory, and kinesthetic [19].

Briefly, auditory learning is a method in which a person learns by listening. Auditory learners depend on listening and speaking as the primary means of learning. Kinesthetic learning directly places learners in situations that closely mirror reality so that they can complete their learning. Visual learning is a style in which the learners use graphics, diagrams, and photographs.

### Intervention description {11a}

#### Pre-inclusion appointment

Patients meeting the selection criteria will be offered the opportunity to participate in the study during the visit prior to the orthodontic appliance placement. The children and their parents will receive oral information about the study and a written information letter.

The free informed and written consent of the child and of both parents will be collected by the investigator before final inclusion in the study. Each patient will be followed for 6 months.

#### Inclusion appointment (D0)

During this visit, the inclusion criteria are checked again. The patient is also randomized to one of the following groups according to the hygiene education technique he will receive. Group 1 (control): the recommendations are given orally, in a chair, by a resident. They are issued during the bonding appointment and at each follow-up appointment. Group 2: the same recommendations as for group 1 are given, completed by a demonstration of dental brushing method at the sink with active participation of the child (more particularly: use of plate developer and then the Oral B electric toothbrush with special orthodontic head; this is done during the bonding appointment and at each inspection appointment). Group 3: the same recommendations as for group 1 are given, but associated with an additional appointment, between the device bonding and the first check-up, i.e., within 15 days of bonding. This is a 15-min session dedicated to teaching oral hygiene. This will include the viewing of an educational video followed by a quiz. Then, an application to the sink is carried out as in group 2.

At the inclusion appointment, the Loë and Silness plaque index (LSPI) and the Loë and Silness gingival inflammation index (GI) will be assessed just prior to placement of the orthodontic appliance by a blind investigator. Both will be performed clinically using a periodontal probe on all 4 sides (buccal, lingual, mesial, and distal) of each maxilar tooth.

At the end of this appointment, each patient will leave with a hygiene kit including an electric toothbrush with two orthodontic heads, Oral B toothpaste, orthodontic wax, and the prescription for pain relief associated with the placement of the appliance.

#### Follow-up appointment (day 0, D45, D90, D135, D180)

Patients in groups 1 and 2 will have 5 appointments (D0, D45, D90, D135, D180). Patients in group 3 will have 6 appointments (D0, between D15, D45, D90, D135, D180).

There will be a check-up appointment (routine practice visits dedicated to archwire changes and the implementation of different mechanics depending on the dysmorphias) every month and a half for 6 months.

During these 4 following check-ups, standardized photographs will be taken after removal of the orthodontic archwires for a later evaluation of the modified orthodontic plaque index (MOP) and the GI will be recorded by an external blinded ratter. After this examination, the oral hygiene advice will be repeated by the resident according to the patient’s randomization group.

### Criteria for discontinuing or modifying allocated interventions {11b}

If biofilm control is very insufficient (generalized visible deposit associated with visible gingival inflammation), the resident will check tooth brushing with the patient. To do this, the patient will brush his/her teeth and then a visual inspection will be made to verify the absence of deposit. This will prevent gum or caries lesions.

### Strategies to improve adherence to interventions {11c}

Patients will only have an oral reminder of the benefit of brushing during follow-up so as not to create a bias.

### Relevant concomitant care permitted or prohibited during the trial {11d}

This research does not require an exclusion period during which the subject cannot participate in another clinical research protocol after the end of the study or after his premature termination.

### Provisions for post-trial care {30}

There are no provisions for post-trial care. After one semester of follow-up, patients normally continue their orthodontic treatments.

### Outcomes {12}

#### Primary outcome

Our main objective is to compare three methods’ effectiveness of oral hygiene education in adolescent patients treated with braces in terms of biofilm control. For that, the average plaque index for the maxillary arch after 6 months of treatment will be measured. The index used is the modified orthodontic plaque index (MOP) [20]. This index will be evaluated on photographs by one of two independent and blind examiners of the patient’s randomization arm. The two examiners (DB and ALF) will have previously calibrated on 10 patients. The calibration will be considered sufficient when the inter-rater reproducibility evaluated by Cohen’s kappa is greater than 0.85.

#### Secondary outcome

The secondary objectives are the effectiveness comparison of the methods for the control of gingival inflammation and the effectiveness comparison of these three methods over time. Two secondary outcomes were chosen. For the evaluation of the control of the inflammation, the average gingival index of Loë and Silness (GI) will be measured 6 months after the installation of the device. For the evaluation of the maintenance of the hygiene control during the treatment, the MOP and the GI scores recorded at each of the control appointments for each group will be compared.

### Participant timeline {13}

**Fig. 2:**
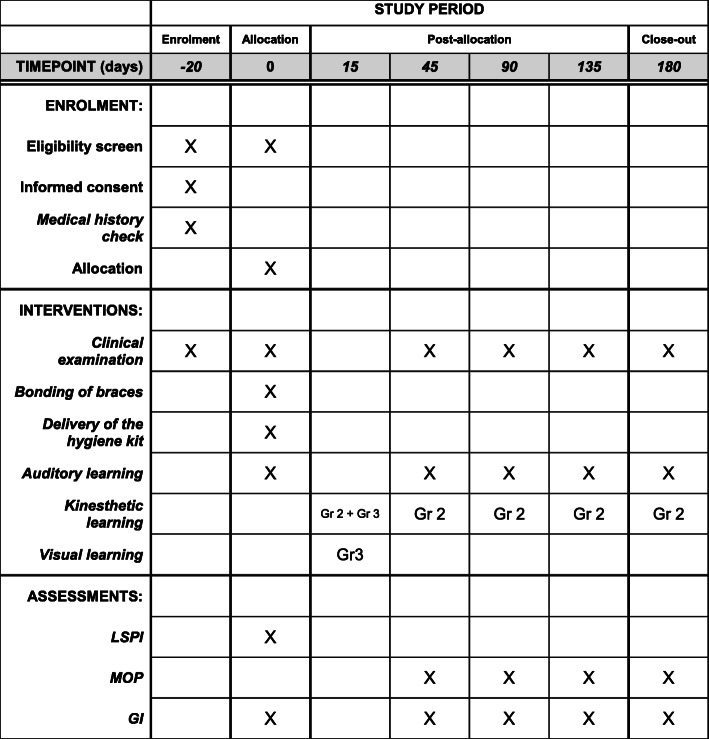
Time schedule of enrolment, interventions, and assessments. Group 1: auditory method, group 2: auditory + kinesthetic method, group 3: auditory + visual method

A time schedule of enrolment, interventions, assessments, and visits for participants is proposed in the form of a diagram (Fig. 2).

### Sample size {14}

The sample size calculation is based on the primary outcome: comparison of the mean MOP between the 3 experimental groups after 6 months of treatment. For a risk alpha = 5%, a power of 80%, an effect size of 0.35, with an ANOVA type analysis and 3 groups, a total of 81 subjects is necessary (27 per group). To consider possible dropouts, we plan to recruit 30 patients per group, i.e., 90 patients in total.

### Recruitment {15}

To achieve adequate participant enrollment to reach the target sample size, it is expected that all residents and practitioners of the department will participate. A total of around 30 bondings is made weekly in the department. It would allow to obtain the complete sample in 3–4 weeks of inclusion.

## Assignment of interventions: allocation

### Sequence generation {16a}

Randomization will be carried out beforehand by a computerized system using RStudio® software version 1.4.1103 (RStudioTeam) with R version 4.0.2 (RCore Team). It will be organized by an independent person. The library used is “blockrand” with the blockrand function. The script is blockrand (*n* = 90, levels = c (“Gr1”, “Gr2”, “Gr3”)). This generates an allocation list in each of the groups equally and randomly.

### Concealment mechanism {16b}

The sequence is generated beforehand by a person external to the study. Then, tickets with mention of the allocation group are printed. They are arranged in the order defined by the sequence. The box containing these ordered tickets will then be taken to the orthodontic department without the practitioners or residents knowing the order of classification.

### Implementation {16c}

The creation of the sequence is carried out. Enrollment is done by residents and practitioners. The assignment to a group is also carried out by the practitioners or residents who treat the patient. When a patient meets the inclusion criteria, the resident who treats him comes to look for a sealed envelope containing the ticket described above. Then, the anonymized identifier and the patient group are collected in a database separate from the rest of the study and stored in the clinical research department of the hospital.

## Assignment of interventions: blinding

### Who will be blinded {17a}

Participants cannot be blinded in this study given the nature of the interventions. Children and their parents will know they are taking part in a trial. The practitioners and the residents who treat patients are not blinded. The photos taken for the evaluation of the primary outcome will be taken by the resident ensuring the treatment of the patient. The evaluators will be blinded because the evaluation of the main outcome is done on these photographs at a distance from the patient by a foreign examiner of the project.

The evaluation of the secondary outcomes is done in the chair by an evaluator external to the project. To avoid bias, the patient is informed not to communicate about his randomization arm and a resident or practitioner will always be present with him to avoid leaks. Moreover, a photographic retractor will be placed in the mouth to limit the dialogue with the patient.

### Procedure for unblinding if needed {17b}

The lifting of the blind will then be able to be carried out by the resident or the practitioner who treats the patient.

## Data collection and management

### Plans for assessment and collection of outcomes {18a}

LSPI will only be used once per patient during the bonding appointment. It will be read with a periodontal probe on the 4 sides of each tooth from 16 to 26 (12 teeth so 48 measurements in total per patient). Then, to evaluate the plaque control, the MOP will be used at each appointment. Its evaluation is done on 3 intra-oral photographs in occlusion after removal of the orthodontic archwires. They will be calibrated using a spreader with green marks to have the same magnification. The camera used is a Nikon D7000 model with an AF-S Micro Nikkor 60-mm lens. The settings used will be manual mode, F32 aperture, shutter speed 200, 1:3 magnification. The photographs will be taken by the resident, but the posterior evaluation will be carried out by one of the two examiners, blind to the allocation of the patient to one of the three groups.

The GI will be evaluated at each appointment and for each patient using a periodontal probe on the four faces of the teeth from 16 to 26 (48 measurements per patient). All clinical measurements will be performed by one of the study’s two raters.

### Plans to promote participant retention and complete follow-up {18b}

Subjects may withdraw their consent and request to exit the study at any time and for any reason. Data prior to this withdrawal of consent will be collected unless the participant objects in writing. The investigator may temporarily or permanently interrupt the participation of a subject in the study for any reason that would best serve the subject’s interests. In the event of a premature exit, the investigator must document the reasons as completely as possible. Leaving a participant’s study should not change their usual care in relation to their illness. In the event of a subject lost to follow-up, the investigator will make every effort to resume contact with the person.

### Data management {19} and confidentiality {27}

All clinical data will be collected in an anonym/individual case report form (CRF). The data will then be compiled in an Excel spreadsheet meeting the requirements of the French Data Protection Authority (FDPA).

The Excel file including the anonymized data and that of the correspondence table will be protected by a password and saved on the secure server of the Rennes hospital. They will not be transmitted outside the establishment. The files will be accessible only to the investigators and examiners. Photo shoots will be kept in the patient’s CRF.

Patient demographic data (age at inclusion, gender) and date of signature of consent are collected. At each visit, the following information is noted: date, caries, plaque index (LSPI or MOP) and gingical index for each tooth, lack of hygiene, demonstration at the sink, detachment of braces, and photographs. During the last visit, the date of close-out from the study is noted and in the event of early close-out, the reason.

### Plans for collection, laboratory evaluation, and storage of biological specimens for genetic or molecular analysis in this trial/future use {33}

Not applicable.

## Statistical methods

### Statistical methods for primary and secondary outcomes {20a}

The statistical analysis will have a descriptive and an inferential part. The descriptive statistical analysis of the quantitative variables will be done by giving for each variable the positional parameters (mean, median, minimum, maximum, first and third quartiles) as well as the dispersion parameters (variance, standard deviation, range, interquartile range). The Gaussian character of the data will be tested by the Shapiro-Wilk test and by QQ plot diagrams. The qualitative variables will be described by giving the contingency and proportions of each modality in the sample.

For the first outcomes (comparisons of final MOP between the 3 groups) and for inflammation outcome (gingival index), analyses will be made either by an analysis of variance (Gaussian case) or by its non-parametric equivalent, namely the Kruskall-Wallis test (non-Gaussian data). For the changes of the MOP and of the LSPI in the 3 groups, analyses will be graphically represented (on average and per individual) and analyzed by mixed models (random patient effect) allowing in particular to study the effect of the interventions over time (introduction of the time × group interaction term).

### Interim analyses {21b}

Not applicable.

### Methods for additional analyses (e.g., subgroup analyses) {20b}

For all these analyses, when the data are not Gaussian, the possibility of transforming the variable (via the Box-Cox method or by a “classical” transformation of the square root or logarithmic type) will be studied before using a non-parametric test.

### Methods in analysis to handle protocol non-adherence and any statistical methods to handle missing data {20c}

The analysis will be carried out with the intention to treat (whatever the subjects benefited from the intervention in their group). Analyses will be performed with the R software in its most up-to-date version at the time of analysis and with all the packages required to perform the analyses.

### Plans to give access to the full protocol, participant-level data, and statistical code {31c}

Upon simple request by email, we will communicate the data collected as well as our analysis method.

## Oversight and monitoring

### Composition of the coordinating center and trial steering committee {5d}

The Rennes University Hospital is the promoter of this clinical trial. It transmits to the National Agency for the Safety of Medicines and Health Products the favorable opinion of the Personal Protection Committee and the summary of the study. The trial steering committee is composed of the investigator (DB) and the representative of the Research and Innovation Department.

### Composition of the data monitoring committee, its role, and reporting structure {21a}

The data will be processed by the two investigators (DB and ALF) of the study, and the signed consents will be retrieved and processed by the representative of the Research and Innovation Department of the Rennes University Hospital.

An independent data and safety monitoring board (DSMB) is monitoring the progress and safety of the trial. The DSMB is independent of the trial and is comprised of two academic dentists, who are experienced in the conduct of clinical trials in dentistry and outside the study, being able to pause the trial to investigate or give suggestions on potential safety issues to improve our design and implement.

### Adverse event reporting and harms {22}

Adverse events, undesirable effects, and incidents will be declared to the various health vigilance circuits applicable to each product or practice concerned (care vigilance, pharmacovigilance, materiovigilance, hemovigilance, cosmetovigilance, etc.) in accordance with the regulation’s laws. It will be specified that the patient is included in a clinical trial and to identify precisely the clinical trial concerned.

### Frequency and plans for auditing trial conduct {23}

The investigators (DB and ALF) and associated persons agree to accept any quality assurance audits carried out by the sponsor of the study as well as the inspections carried out by the competent authority and the DSMB. All data, documents, and reports can be subject to regulatory audits and inspections without medical confidentiality being enforceable. The auditing is done randomly and at least once a month.

### Plans for communicating important protocol amendments to relevant parties (e.g., trial participants, ethical committees) {25}

Any substantial modification to the study protocol would be notified to the Personal Protection Committee in order to verify that the proposed modifications do not affect the guarantees given to people who lend themselves to research. A substantial change to the protocol by the investigators must be approved by the promoter. The latter must obtain, prior to the implementation of this modification, a favorable opinion from the Personal Protection Committee. If necessary, a new consent from the people participating in the research will be collected. Any deviations from the protocol will be fully documented using a breach report form, and all updates of the protocol will be communicated to the promoter.

### Dissemination plans {31a}

As a reminder, the objective of this study is to compare three teaching methods for oral hygiene education. This is part of a particularly vague context on communication with young patients. The aim of this project is to provide clinicians with recommendations for good practice in terms of motivation methodology and hygiene education. The ideal is to minimize white spots and gum damages.

The teaching method that has obtained the best results will be presented to the medical community through several ways. We plan to use the communication media of the Oral B company and also to inform orthodontists during congresses.

## Discussion

Mastering oral hygiene during orthodontic treatment is a challenge. The iatrogenic effects of this treatment are carious lesions and gum disease. More than 1 out of 2 patients treated with braces encounter these problems. Beyond the harm suffered by the patient, their care in the USA represents an annual cost of five hundred million dollars. Initial education and motivation for hygiene is therefore a key factor in the success of the treatment. However, regarding the literature, many preventive strategies have been developed to optimize oral hygiene during treatment. But study protocols are at high risk of bias: problems with randomization, maintenance of blindness, and short-term follow-up [16, 17]. Furthermore, most of the publications relating to this subject do not focus on the educational methods to be employed but rather on the equipment and prophylactic measures provided by the orthodontist. Therefore, the clinician does not currently have any reliably established guidelines as to the educational approach to be used for hygiene education. The aim of this study is therefore to standardize the practices in terms of hygiene education during multi-bracket treatment. The main objective of this clinical trial is to compare the effectiveness of three oral hygiene education methods in adolescents treated with braces regarding biofilm control. The trial will evaluate the hypothesis that the plaque index after 6 months of treatment will be the lowest in the visual + kinesthetic learning method group.

To meet this objective, several outcomes can be used. An ideal index should be simple, reliable, economic, and quick to record. There are two types of index: those that quantify the biofilm accumulation and those that assess the inflammatory state of the gingiva. We chose the plaque index which is a very often used outcome in clinical studies focusing on hygiene in orthodontics [21, 22]. The Silness and Loë plaque index is the most widely used in clinical studies although it is not sufficient in orthodontic patients because the plaque accumulation scheme is different [20]. Several studies have therefore proposed indices dedicated to orthodontics [23–25]. In addition, among these indices, none has sufficient reproducibility. To get around this problem, the literature concluded that the use of indices on photographs would therefore be a very interesting alternative [25, 26].

The MOP represents a hybrid of photographic orthodontic indices and traditional dental plaque indices. The advantages of a photographic index are that it is a permanent record and it can be assessed at leisure and be viewed on multiple occasions, enabling assessment of reproducibility [27]. Besides, regarding the inter- and intra-examiner reliability, the MOP is the most reproducible. Another advantage is that the MOP is graded with a high subjective validity because it offers greater discrimination of the gingival third. Taken together, these elements make the discrimination performance of the MOP excellent [27]. The MOP enables the examiner to assess the plaque accumulation at the gingival margin and around the bracket. Scores from 0 to 4 are assigned: 0 indicates no plaque; 1 indicates inter-proximal plaque accumulation (mesial and/or distal) of the bracket base; 2 indicates plaque accumulation inter-proximal, incisal, and/or cervical to the bracket base; 3 indicates continuous plaque accumulation from the gum line to the bracket base; and 4 indicates complete coverage by plaque. For 3 and 4, the plaque is not isolated to one of the two regions described by this index. The study analyzes only the maxillary teeth because taking the photographic image would have been complicated in the mandible. Anterior overbite would have made open-mouth photographs necessary. This significantly complicated the process and increased the risk of bias. In addition, it is common to bond the arches at different times. This makes measurements more complicated with an increased risk of error during measurements.

The GI often appears as a secondary outcome. In fact, the most frequently used is the Loë and Silness index. It varies with the general condition of the patient and does not only consider the bonded dental surfaces. For this reason, it is used for the secondary outcome.

Several learning styles are compared in this study. Learning styles refer to a range of theories aimed at explaining individuals’ differences in learning. Barbe and Swassing proposed three learning methods identified by the acronym VAK: visual, auditory, and kinesthetic. The learning modality forces can occur independently or in combination [19].

Auditory learning is a method in which a person learns by listening. A hearing learner depends on listening and speaking as the primary means of learning. In group 1, four items are orally presented in 2 min. They relate to the definition of dental plaque, the value of brushing after each meal, the principle of brushing with an electric brush, and bleeding gums and their management.

Kinesthetic learning is quite close to the experiential learning cycle strategy described by Kolb. It focuses on the learning process rather than on results and directly places learners in situations that reflect reality as closely as possible so that they can carry out their learning. These learners are fully involved in their learning process and feel responsible for their actions [28]. It adds a stage of realization to the sink. To do this, the GC Tri Plaque ID Gel® developer is deposited on the teeth and then the patient brushes his teeth using the modified Bass technique. This method is applied for group 2.

Visual learning is a style in which the learner uses graphics, diagrams, and photographs. In group 3, visual learning in addition to the kinesthetic learning consists of viewing a film during a dedicated session. It shows what the plaque is, then it shows the consequences of poor brushing with examples. Finally, the film gives the patient the keys to know how to brush their teeth and assess whether brushing is sufficient. Knowledge is then assessed by a quiz [29–31]. For all methods, excepting the film, the information is repeated at each session; this promotes learning [30, 31]. Most studies that have focused their efforts on visual learning have found that visual learning styles, as opposed to traditional learning styles, greatly improve the whole adolescent learning experience. Visual learning engages adolescents. This is one of the most important factors for teens to be motivated to learn. The interest of adolescents is increased thanks to the use of graphic and video animation [30, 32, 33].

As this study relates to the educational technics and not prophylactic, to avoid creating bias, all patients will be offered the same dental hygiene kit. This consists of an electric toothbrush, special heads for orthodontics, and toothpaste. The choice fell on the electric method because the literature shows its superiority to clean the teeth which wear braces [23, 34].

## Trial status

The study protocol, version 1.1, 05 June 2020, has been approved by the research ethics committee of Bordeaux’s hospital. The trial will be conducted following the principles of the Declaration of Helsinki and is registered with the ClinicalTrials.gov under number NCT04444154, registration date 22 June 2020.

Patient recruitment will begin in January 2021 and end in September 2021.

## Abbreviations

MOP: Modified orthodontic plaque index
GI: Loë and Silness gingival inflammation index
LSPI: Loë and Silness plaque index

## References

[1] OMS. Préambule de la constitution de l’OMS [Internet]. 1946 [cited 2019 Jul 9]. Available from: https://www.who.int/fr/about/who-we-are/constitution.

[2] Sischo L, Broder HL (2011). Oral health-related quality of life. J Dent Res.

[3] Herkrath APCQ, Vettore MV, de Queiroz AC, Alves PLN, Leite SDC, Pereira JV, et al. Orthodontic treatment need, self-esteem, and oral health-related quality of life among 12-yr-old schoolchildren. Eur J Oral Sci. 2019;0 Available from: http://onlinelibrary.wiley.com/doi/abs/10.1111/eos.12611. [cited 2019 May 3]. 10.1111/eos.12611 30891853

[4] Palomares NB, Celeste RK, de Oliveira BH, Miguel JAM (2012). How does orthodontic treatment affect young adults’ oral health-related quality of life?. Am J Orthod Dentofac Orthop Off Publ Am Assoc Orthod Its Const Soc Am Board Orthod..

[5] van Gastel J, Quirynen M, Teughels W, Coucke W, Carels C (2011). Longitudinal changes in microbiology and clinical periodontal parameters after removal of fixed orthodontic appliances. Eur J Orthod.

[6] Hadler-Olsen S, Sandvik K, El-Agroudi MA, Øgaard B (2012). The incidence of caries and white spot lesions in orthodontically treated adolescents with a comprehensive caries prophylactic regimen--a prospective study. Eur J Orthod.

[7] Zachrisson BU (1976). Cause and prevention of injuries to teeth and supporting structures during orthodontic treatment. Am J Orthod.

[8] Mei L, Busscher HJ, van der Mei HC, Chen Y, de Vries J, Ren Y (2009). Oral bacterial adhesion forces to biomaterial surfaces constituting the bracket-adhesive-enamel junction in orthodontic treatment. Eur J Oral Sci.

[9] Øilo M, Bakken V (2015). Biofilm and dental biomaterials. Materials..

[10] Lundström F, Krasse B (1987). Streptococcus mutans and lactobacilli frequency in orthodontic patients; the effect of chlorhexidine treatments. Eur J Orthod.

[11] Antonelli G, Clementi M, Pozzi G, Rossolini GM. Principi di microbiologia medica. Italy: CEA.

[12] Lucchese A, Bondemark L, Marcolina M, Manuelli M (2018). Changes in oral microbiota due to orthodontic appliances: a systematic review. J Oral Microbiol.

[13] Wong BKJ, McGregor NR, Butt HL, Knight R, Liu LY, Darby IB (2016). Association of clinical parameters with periodontal bacterial haemolytic activity. J Clin Periodontol.

[14] Geiger AM, Gorelick L, Gwinnett AJ, Benson BJ (1992). Reducing white spot lesions in orthodontic populations with fluoride rinsing. Am J Orthod Dentofac Orthop Off Publ Am Assoc Orthod Its Const Soc Am Board Orthod..

[15] Ren Y, Jongsma MA, Mei L, van der Mei HC, Busscher HJ (2014). Orthodontic treatment with fixed appliances and biofilm formation--a potential public health threat?. Clin Oral Investig.

[16] Derks A, Katsaros C, Frencken JE, van’t Hof MA, Kuijpers-Jagtman AM (2004). Caries-inhibiting effect of preventive measures during orthodontic treatment with fixed appliances. A systematic review. Caries Res.

[17] Benson PE, Parkin N, Dyer F, Millett DT, Furness S, Germain P. Fluorides for the prevention of early tooth decay (demineralised white lesions) during fixed brace treatment. Cochrane Database Syst Rev. 2013;12:CD003809. 10.1002/14651858.CD003809.pub3 24338792

[18] Derks A, Kuijpers-Jagtman AM, Frencken JE, Van’t Hof MA, Katsaros C (2007). Caries preventive measures used in orthodontic practices: an evidence-based decision?. Am J Orthod Dentofac Orthop Off Publ Am Assoc Orthod Its Const Soc Am Board Orthod..

[19] Barbe WB, Swassing RH (1979). Teaching through modality strength concepts and practices.

[20] Al-Anezi SA, Harradine NWT (2012). Quantifying plaque during orthodontic treatment. Angle Orthod.

[21] Migliorati M, Isaia L, Cassaro A, Rivetti A, Silvestrini-Biavati F, Gastaldo L (2015). Efficacy of professional hygiene and prophylaxis on preventing plaque increase in orthodontic patients with multibracket appliances: a systematic review. Eur J Orthod.

[22] Huang J, Yao Y, Jiang J, Li C (2018). Effects of motivational methods on oral hygiene of orthodontic patients: a systematic review and meta-analysis. Medicine (Baltimore).

[23] Clerehugh V, Williams P, Shaw WC, Worthington HV, Warren P (1998). A practice-based randomised controlled trial of the efficacy of an electric and a manual toothbrush on gingival health in patients with fixed orthodontic appliances. J Dent.

[24] Costa MR, Silva VC, Miqui MN, Sakima T, Spolidorio DMP, Cirelli JA (2007). Efficacy of ultrasonic, electric and manual toothbrushes in patients with fixed orthodontic appliances. Angle Orthod.

[25] Thienpont V, Dermaut LR, Van Maele G (2001). Comparative study of 2 electric and 2 manual toothbrushes in patients with fixed orthodontic appliances. Am J Orthod Dentofac Orthop Off Publ Am Assoc Orthod Its Const Soc Am Board Orthod..

[26] Klukowska M, Bader A, Erbe C, Bellamy P, White DJ, Anastasia MK (2011). Plaque levels of patients with fixed orthodontic appliances measured by digital plaque image analysis. Am J Orthod Dentofac Orthop Off Publ Am Assoc Orthod Its Const Soc Am Board Orthod..

[27] Smith RN, Brook AH, Elcock C (2001). The quantification of dental plaque using an image analysis system: reliability and validation. J Clin Periodontol.

[28] Kolb DA (1984). Experiential learning: Experience as the source of learning and development.

[29] Peng Y, Wu R, Qu W, Wu W, Chen J, Fang J (2014). Effect of visual method vs plaque disclosure in enhancing oral hygiene in adolescents and young adults: a single-blind randomized controlled trial. Am J Orthod Dentofac Orthop Off Publ Am Assoc Orthod Its Const Soc Am Board Orthod..

[30] Lees A, Rock WP (2000). A comparison between written, verbal, and videotape oral hygiene instruction for patients with fixed appliances. J Orthod.

[31] Boyd RL, Chun YS (1994). Eighteen-month evaluation of the effects of a 0.4% stannous fluoride gel on gingivitis in orthodontic patients. Am J Orthod Dentofac Orthop Off Publ Am Assoc Orthod Its Const Soc Am Board Orthod.

[32] McNab M, Skapetis T (2019). Why video health education messages should be considered for all dental waiting rooms. PLoS One.

[33] Moshkelgosha V, Mehrvarz S, Saki M, Golkari A (2017). Computer-based oral hygiene instruction versus verbal method in fixed orthodontic patients. J Dent Biomater.

[34] Schätzle M, Sener B, Schmidlin PR, Imfeld T, Attin T (2010). In vitro tooth cleaning efficacy of electric toothbrushes around brackets. Eur J Orthod.

